# 6,7,4′-Trihydroxyflavanone Mitigates Methamphetamine-Induced Neurotoxicity in SH-SY5y Cells via Nrf2/heme Oxyganase-1 and PI3K/Akt/mTOR Signaling Pathways

**DOI:** 10.3390/molecules26092442

**Published:** 2021-04-22

**Authors:** Hyun-Su Lee, Gil-Saeng Jeong

**Affiliations:** College of Pharmacy, Keimyung University, Daegu 42601, Korea; hyunsu.lee@kmu.ac.kr

**Keywords:** methamphetamine, 6,7,4′-trihydroxyflavanone, Nrf2, HO-1, PI3K, neurotoxicity, SH-SY5y cells

## Abstract

Methamphetamine (METH) is a synthetic psychostimulant drug that has detrimental effects on the health of its users. Although it has been investigated as a cause of neurodegenerative disease due to its neurotoxicity, whether small molecules derived from natural products attenuate these side effects remains elusive. 6,7,4′-trihydroxyflavanone (THF) is a flavanone family that possesses various pharmacological activities, including anti-rheumatic, anti-ischemic, anti-inflammatory, anti-osteoclastogenic, and protective effects against METH-induced deactivation of T cells. However, little is known about whether THF protects neuronal cells from METH-induced neurotoxicity. Here, we investigated the protective effects of THF on neurotoxicity induced by METH exposure by enhancing the Nrf2/HO-1 and PI3K/Akt/mTOR signaling pathways in SH-SY5y cells. Treatment with THF did not lead to cytotoxicity, but attenuated METH-induced neurotoxicity by modulating the expression of apoptosis-related proteins, METH-induced oxidative stress, and PI3K/Akt/mTOR phosphorylation in METH-exposed SH-SY5y cells. Moreover, we found THF induced Nrf2 nuclear translocation and HO-1 expression. An inhibitor assay confirmed that the induction of HO-1 by THF attenuates METH-induced neurotoxicity. Therefore, we suggest that THF preserves neuronal cells from METH-induced neurotoxicity by upregulating HO-1 expression through the Nrf2 and PI3K/Akt/mTOR signaling pathways. Thus, THF has therapeutic potential for use in the treatment of METH-addicts suffering from neurodegenerative diseases.

## 1. Introduction

Methamphetamine (METH) is a highly addictive drug whose abuse has caused significant social and public health issues for decades [1]. It has been shown to activate neuronal cells to release neurotransmitters, promoting hallucinations and euphoria [2]. Various studies have reported that repeated exposure to METH causes severe neurotoxicity, which induces neuronal cell death [3,4]. In addition, accumulating evidences from animal experiments have shown that neuronal toxicity by METH exposure leads to the pathogenesis of neurodegenerative diseases, including Alzheimer’s disease and Parkinson’s disease [5,6]. A chronic effect of METH on neurodegenerative diseases has been shown to be associated with the production of reactive oxygen species (ROS) and damage to cellular molecules, including proteins, DNA, and lipids in neuronal cells [7,8]. Therefore, the removal of accumulating oxidative stress induced by METH exposure using antioxidants may be a promising strategy to attenuate neurotoxicity.

Redox-sensitive transcription factors are known to play a critical role in the defense against oxidative stress in the brain. Nuclear factor erythroid 2-related factor 2 (Nrf2), one of most important factors for cytoprotection, has been reported to be activated in the stimulation of small molecules, including epigallocatechin-3-gallate (EGCG), thymoquinone, and vitamin D2 [9,10,11,12]. Inactive Nrf2 forms in the cytosol via a heterodimer and its repressor Kelch-like ECH associating protein 1 (keap1). However, stimulants lead to the nuclear translocation of Nrf2 into the nucleus in dissociation with keap1, where it acts as a transcription factor [13]. It has also been found that Nrf2 transcribes genes of cytoprotective enzymes, including heme oxygenase1 (HO-1), NAD(P)H:quinone oxidoreductase (NQO1), and oxoguanine glycosylase 1 (OGG1) [14,15,16]. In particular, HO-1 is a cytoprotective enzyme that protects cells from apoptotic, autophagic, and hypoxia-induced necrotic pathways via the regulation of oxidative stress [17,18,19]. Phosphatidylinositol 3-kinase (PI3K)/Akt/mTOR pathway has been elucidated to play a critical role for cell survival, anti-apoptosis, proliferation, and cell growth [20]. Phosphorylation levels of PI3K/Akt/mTOR are downregulated by various apoptotic stimuli including METH that cause cell death or damages [21]. Although METH exposure triggers oxidative stress, generated ROS suppresses the phosphorylation of the PI3K/Akt/mTOR signaling pathway in neuronal cells [22]; however, whether small molecules derived from natural products protect cells from METH-induced neurotoxicity by enhancing the Nrf2/HO-1 signaling pathway is yet to be demonstrated.

6,7,4′-trihydroxyflavanone (THF, C_15_H_12_O_5_), a bioactive molecule of the flavanone family, is isolated from *Dalbergia odorifera* (Leguminosae) [23] and has been traditionally used for its anti-epigastric pain, anti-rheumatic, anti-ischemic, and anti-swelling properties [24]. Recent reports have shown that THF possesses several bioactivities against osteoclastogenesis in bone formation, exerting anti-inflammatory effects against dextran sodium sulfate (DSS)-induced immune bowel disease (IBD) and protective effects against hypoxia-induced neurotoxicity via the enhancement of the Nrf2/HO-1 signaling pathway [23,25]. In particular, the ameliorative effect of THF on METH-induced cytotoxicity has been studied in T cells, where pretreatment with THF significantly restored the METH-induced deactivation of T cells under stimulated conditions [26]. Although the beneficial bioactivities of THF have been elucidated in several fields, little is known about the protective effect of THF against METH-induced neurotoxicity in neuronal cells and its underlying mechanism of action. In the present study, we evaluated the attenuating effect of THF on METH-induced neurotoxicity using SH-SY5y neuronal cells. To understand the underlying mechanism, we examined whether THF induced HO-1 through the Nrf2 and PI3K/Akt/mTOR signaling pathways.

## 2. Results

### 2.1. Treatment with THF Does Not Have a Negative Effect on SH-SY5y Neuronal Cells

**Figure 1:**
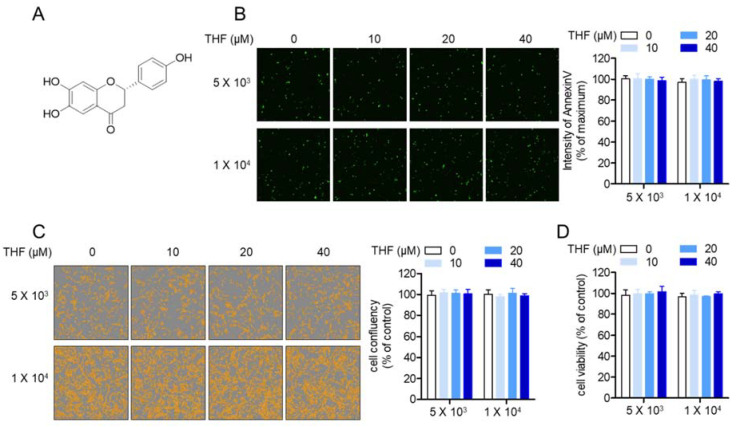
(**A**) The chemical structure of 6,7,4′-trihydroxyflavanone (THF). (**B**–**D**) SH-SY5y cells (5 × 10^3^/well (top) or 1 × 10^4^/well (bottom), 96-well plate) were stained with 1× annexin V and 1× caspase 3/7 staining reagent and then incubated with the indicated concentrations of THF for 24 h. After incubation, the intensity of annexin V (**B**) and cell confluency (**C**) was determined using the IncuCyte imaging system. For the measurement of cell viability, the MTT assay was conducted and the percentage of the control was calculated based on the resulting OD value (**D**). Results are shown as the mean value of three experiments ± SEM.

Following from a previous study from our group in which the treatment of SH-SY5y cells at a density of 1 × 10^4^/well with THF (Figure 1A) was not cytotoxic [27], we investigated whether THF leads to apoptosis in SH-SY5y cells at a lower density. Figure 1B shows that THF up to a concentration of 40 μM did not induce cytotoxicity in SH-SY5y cells at densities of 5 × 10^3^/well or 1 × 10^4^/well. Cell confluent images obtained from the IncuCyte imaging system analysis showed that treatment with THF is not involved in cell arrest in proliferation up to a concentration of 40 μM (Figure 1C). To confirm whether THF was non-cytotoxic, an MTT assay was performed. Figure 1D shows that treatment with THF did not have any negative effects on SH-SY5y cells at densities of 5 × 10^3^/well or 1 × 10^4^/well.

### 2.2. THF Attenuates METH-Induced Neurotoxicity in SH-SY5y Neuronal Cells

**Figure 2:**
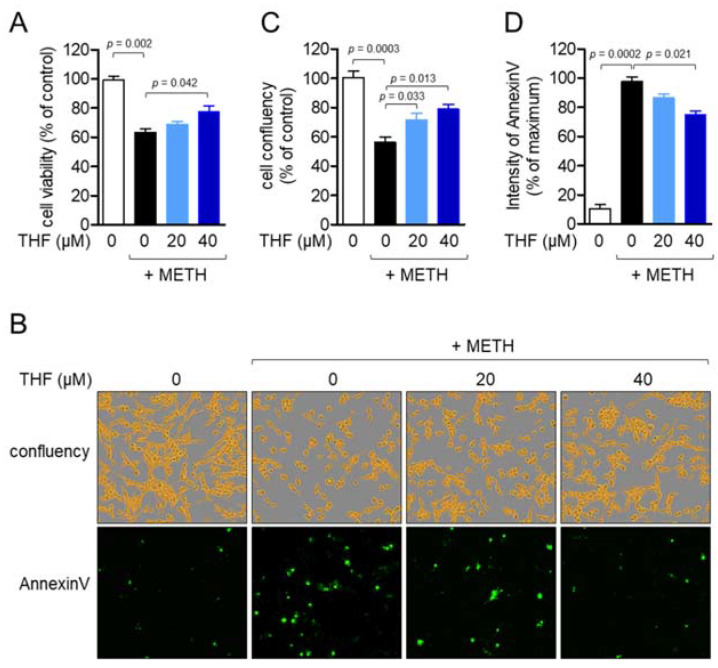
(**A**) SH-SY5y cells (1 × 10^4^/well, 96-well plate) were pretreated with the indicated concentration of THF for 6 h and then exposed to 2 mM methamphetamine (METH) for an additional 24 h. Cell viability was assessed by the methyl thiazol tetrazolium (MTT) assay and the percentage of the control was calculated based on the resulting OD value. (**B**–**D**) SH-SY5y cells (1 × 10^4^/well, 96-well plate) were stained with 1× annexin V staining reagent and then pretreated with the indicated concentration of THF for 6 h. The cells were exposed to 2 mM METH for 24 h, and fluorescent images were obtained using the IncuCyte imaging system (**B**). Adherent cells are shown in yellow. (**C**,**D**) Confluency (**C**) and the intensity of annexin V (**D**) were calculated and presented as the percentage of the control for (**C**) and the percentage of the maximum for (**D**), respectively. Results are shown as the mean value of three experiments ± SEM in the bar graph. *p* value between two indicated groups.

To understand whether pretreatment with THF reduced the cytotoxicity of SH-SY5y cells induced by METH exposure, the MTT cell viability assay was performed in a dose-dependent manner. The concentration of METH was selected by comparing to the previous study and MTT assay (Supplementary Figure S1). While exposure to 2 mM METH significantly mitigated cell viability, pretreatment with THF protected SH-SY5y cells from METH-induced cytotoxicity (Figure 2A). To determine whether the METH-induced apoptotic pathway was inhibited by pretreatment with THF in SH-SY5y cells, the intensity of annexin V was assessed. The cellular confluency of SH-SY5y cells was restored by pretreatment with THF in a dose-dependent manner (Figure 2B,C). Figure 2B,D show that pretreatment with THF regulates the intensity of annexin V induced by METH exposure in SH-SY5y cells. These data suggest that METH leads to cytotoxicity in SH-SY5y cells, while pretreatment with THF significantly attenuates its cytotoxicity.

### 2.3. THF Modulates the Expression of Apoptosis-Related Proteins in METH-Exposed Condition

**Figure 3:**
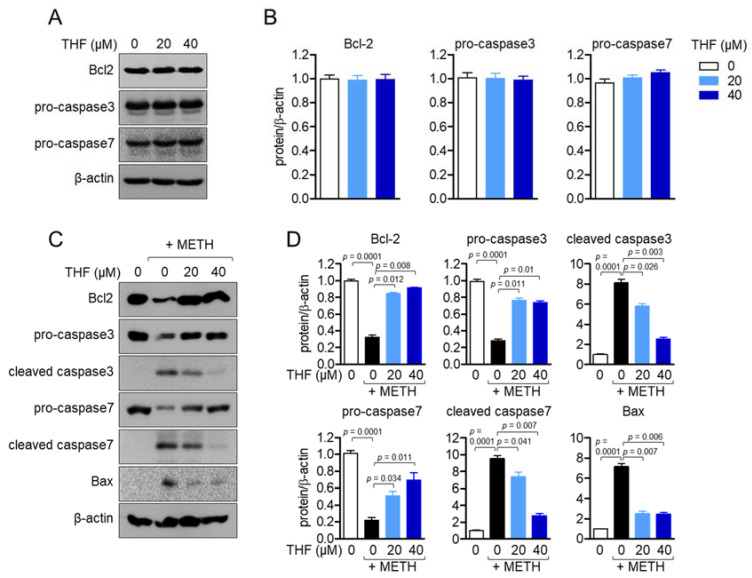
(**A**) SH-SY5y cells were treated with the indicated concentration of THF for 24 h and the expressions of the indicated proteins were detected by western blot. (**B**) The indicated proteins were normalized with the level of β-actin and presented in a bar graph. (**C**) SH-SY5y cells were pretreated with the indicated concentration of THF for 6 h and then exposed to 2 mM METH for an additional 24 h. The expression of apoptosis-related proteins was detected using western blot analysis. (**D**) The indicated proteins were normalized with the level of β-actin and presented in a bar graph. Results are shown as the mean value of three experiments ± SEM. *p* value between two indicated groups.

Since the apoptotic pathway induced by METH exposure is highly involved in the expression of apoptosis-related proteins, including Bcl2, caspase family, and Bax, we firstly examined whether THF treatment leads to the changes of apoptosis-related proteins expression. Figure 3A,B showed that THF treatment up to 40 μM did not alter the expressions of apoptosis-related proteins. We investigated whether pretreatment with THF affects the expression of these proteins. Figure 3C,D revealed that METH exposure significantly reduced the expression of Bcl2 and led to the cleavage of the caspase family, but induced the expression of Bax, a pro-apoptotic protein. Interestingly, pretreatment with THF effectively preserved the effect of METH on SH-SY5y cells in a dose-dependent manner. These results suggest that METH exposure induces the apoptotic pathway by affecting apoptosis-related proteins, while pretreatment with THF restores the effect of METH on SH-SY5y cells.

### 2.4. THF Regulates METH-Induced Oxidative Stress in SH-SY5y Cells

**Figure 4:**
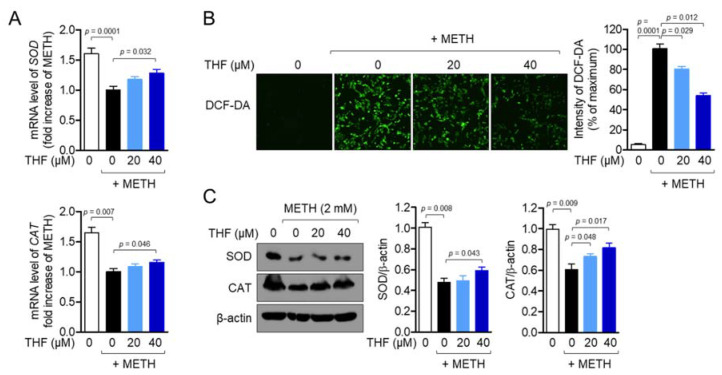
(**A**) SH-SY5y cells were pretreated with the indicated concentration of THF for 6 h and then exposed to 2 mM METH for an additional 6 h. The mRNA levels of superoxidase (*SOD)* and catalase (*CAT)* genes were determined using real-time PCR analysis. (**B**) SH-SY5y cells (1 × 10^4^/well, 96-well plate) were pretreated with the indicated concentration of THF for 6 h and exposed to 2 mM for an additional 24 h. After incubation, the cells were stained with 2 μM 2′,7′-dichlorofluorescin diacetate (DCF-DA) for 20 min. The resulting production of reactive oxygen species (ROS) was detected using the IncuCyte imaging system. The representative images were obtained and the intensity of DCF-DA was calculated as the percentage of the maximum. (**C**) SH-SY5y cells were pretreated with the indicated concentration of THF for 6 h and then exposed to 2 mM METH for an additional 24 h. The expression of SOD and CAT was detected by western blot analysis. The indicated proteins were normalized with the level of β-actin and presented in a bar graph. Results are shown as the mean value of three experiments ± SEM. *p* value between two indicated groups.

METH exposure has been shown to cause cytotoxicity by inducing oxidative stress in SH-SY5y cells [28]. To determine whether pretreatment with THF attenuates oxidative stress induced by METH exposure in SH-SY5y cells, we first determined the mRNA levels of superoxide (SOD) and catalase (CAT) in SH-SY5y cells exposed to METH. As shown in Figure 4A, METH exposure suppressed the mRNA levels of SOD and CAT in SH-SY5y cells. However, pretreatment with THF reversed these effects in a dose-dependent manner. To determine whether ROS production is regulated by pretreatment with THF in SH-SY5y cells, ROS generation was detected by staining the cells with 2′,7′-dichlorofluorescin diacetate (DCF-DA). METH exposure significantly increased the production of ROS in SH-SY5y cells, while THF pretreatment downregulated ROS production in a dose-dependent manner (Figure 4B). To confirm whether pretreatment with THF restores the expression of SOD and CAT in METH-exposed SH-SY5y cells, we performed a western blot assay after pretreatment with THF and exposure to METH. Figure 4C shows that the expression of SOD and CAT was preserved by pretreatment with THF. These results suggest that METH exposure induces oxidative stress, while pretreatment with THF partially blocks it by enhancing the expression of antioxidative genes, including SOD and CAT, on mRNA level and protein level.

### 2.5. THF Restores PI3K/Akt/mTOR Phosphorylation in METH-Exposed SH-SY5y Cells

**Figure 5:**
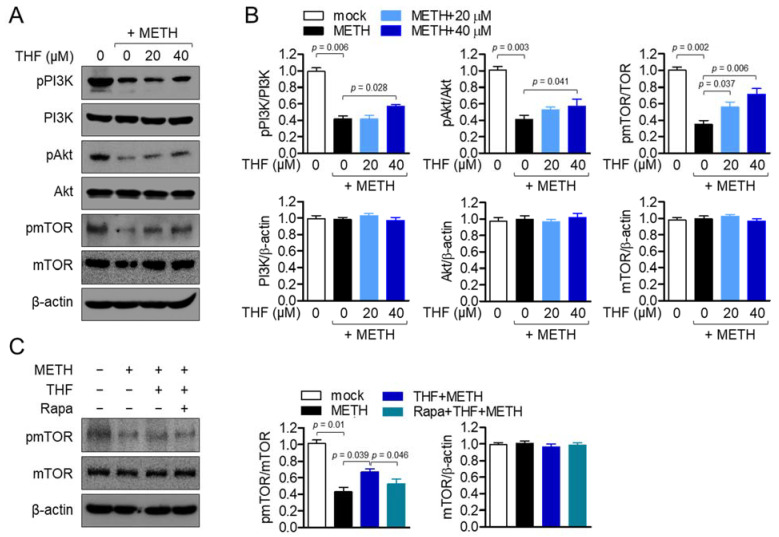
(**A**) SH-SY5y cells were pretreated with the indicated concentration of THF for 6 h and then exposed to 2 mM METH for an additional 1 h. The expression levels of phosphorylated phosphoinositide 3-kinase (PI3K), Akt, and mammalian target of rapamycin (mTOR) were detected by western blot analysis. (**B**) The phosphorylation levels were normalized to the level of total protein and total proteins were normalized with the level of β-actin. (**C**) SH-SY5y cells were pretreated with 50 μM rapamycin for 1 h and then 40 μM of THF for additional 1 h. Cells were exposed to 2 mM METH for 30 min for western blot. The level of phosphorylated mTOR, total mTOR and β-actin were detected and normalized with the loading control. Results are shown as the mean value of three experiments ± SEM. *p* value between two indicated groups.

The PI3K/Akt/mTOR signaling pathway has been shown to play a critical role in cell survival [29]. METH exposure has been reported to induce cytotoxicity through the Pi3K/Akt/mTOR signaling pathway in SH-SY5y cells [30]. As such, the phosphorylation level of PI3K was investigated. Figure 5A,B shows that PI3K phosphorylation was suppressed by METH exposure, while pretreatment with THF restored it in SH-SY5y cells. To determine whether pretreatment with THF affects the downstream PI3K signaling pathway, the phosphorylation levels of Akt and mTOR were evaluated. Although phosphorylated Akt and mTOR were found to be regulated by METH exposure, this effect was reversed by pretreatment with THF in SH-SY5y cells. To elucidate whether protective effect of THF on neurotoxicity is through PI3K/Akt/mTOR pathway, we performed inhibitor assay using rapamycin, which is a well-known inhibitor of mTOR. Figure 5C revealed that pretreatment with rapamycin significantly abrogated the restored phosphorylation of mTOR by pretreatment with THF. These findings suggest that METH exposure controls the phosphorylation levels of the PI3K/Akt/mTOR signaling pathway, while pretreatment with THF restores METH-exposed SH-SY5y cells.

### 2.6. THF Induces Nrf2 Nuclear Translocation and HO-1 Expression in SH-SY5y Cells

**Figure 6:**
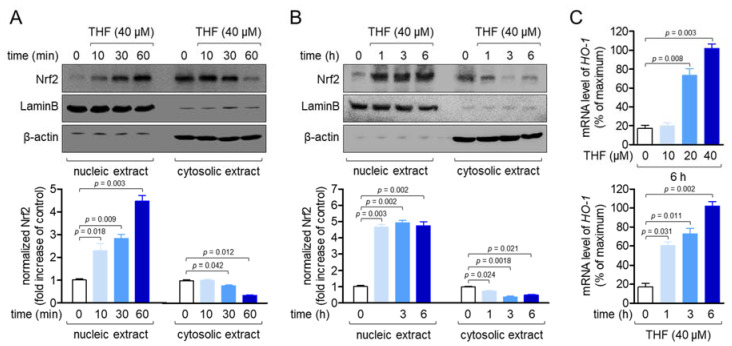
(**A**,**B**) SH-SY5y cells were treated with 40 µM THF for the indicated time (0 to 60 min for (**A**) and 0 to 6 h for (**B**)). The nuclear extract was separated from the whole lysate using a NE-PER kit to evaluate Nrf2 translocation by western blot analysis. The nucleic extract was normalized to the level of LaminB, while cytosolic extract was normalized with the level of β-actin. Normalized Nrf2 is presented as a fold increase of the control. (**C**) SH-SY5y cells were treated with the indicated concentration of THF for 6 h (top) or 40 μM for the indicated time, and the mRNA levels of the HO-1 genes were determined by real-time PCR analysis. The mRNA levels of HO-1 were normalized to the levels of *GAPDH* and presented in percentage of maximum. Results are shown as the mean value of three experiments ± SEM. *p* value between two indicated groups.

The Nrf2/HO-1 signaling pathway is one of the most effective underlying mechanisms for the protection of cells against METH-induced cytotoxicity by small molecules [22,31]. In a recent study, our group reported that THF promotes the Nrf2/HO-1 pathway in SH-SY5y cells in a dose-dependent manner [27]. As such, we examined whether THF leads to the nuclear translocation of Nrf2 in a time-dependent manner. As shown in Figure 6A, the nuclear translocation of Nrf2 from the cytosol increased gradually, and cytosolic Nrf2 was downregulated within 1 h. We also confirmed that nuclear translocation of Nrf2 was maintained up to 6 h post-treatment with THF (B). To determine whether treatment with THF has a cytoprotective effect, the mRNA levels of *HO-1*, which protects cells from toxicity, were analyzed by quantitative PCR analysis. Figure 6C shows that THF treatment induced the mRNA levels of HO-1 in a time-and dose-dependent manner. These data suggest that THF induces the Nrf2/HO-1 signaling pathway to exert cytoprotective effects on SH-SY5y cells.

### 2.7. Induction of HO-1 by THF Treatment Attenuates METH-Induced Neurotoxicity

**Figure 7:**
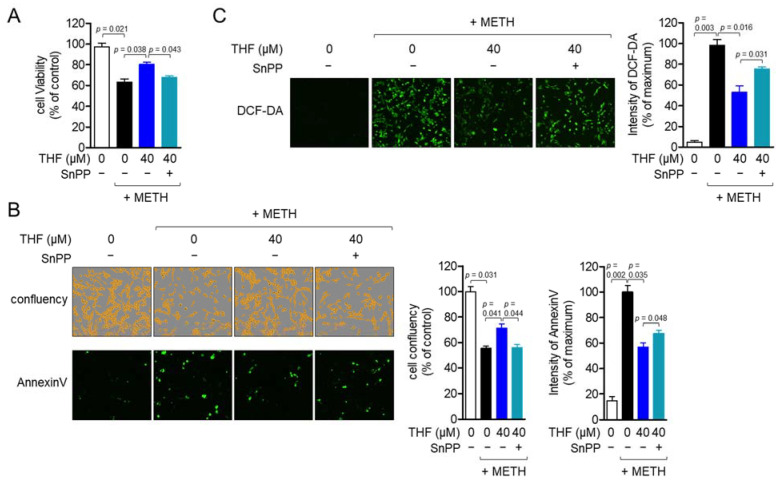
(**A**) SH-SY5y cells were pretreated with 20 μM tin-protoporphyrin IX (SnPP) for 1 h and then pretreated with 40 μM THF for an additional 6 h. After pretreatment, the cells were exposed to 2 mM METH for an additional 24 h. The cell viability was assessed by the MTT assay and the percentage of the control was calculated based on the resulting OD value. (**B**) SH-SY5y cells (1 × 10^4^/well, 96-well plate) were stained with 1× annexin V and 1× caspase 3/7 staining reagent and then pretreated with 20 μM SnPP for 1 h. The cells were additionally pretreated with 40 μM THF for 6 h and then exposed to 2 mM METH for an additional 24 h. Fluorescent images were obtained using the IncuCyte imaging system, and the representative images are presented. The cell confluency and intensity of annexin V and caspase 3/7 were calculated and are presented as a percentage of the control. (**C**) SH-SY5y cells (1 × 10^4^/well, 96-well plate) were pretreated with 20 μM SnPP for 1 h and then pretreated with 40 μM THF for 6 h. After pretreatment, the cells were exposed to 2 mM METH for 24 h. The cells were stained with 2 μM DCF-DA for 20 min and the resulting ROS production was detected using the IncuCyte imaging system. The representative images were obtained and the intensity of DCF-DA was calculated as a percentage of the maximum. Results are shown as the mean value of three experiments ± SEM. *p* value between two indicated groups.

Treatment with THF was found to induce the Nrf2/HO-1 signaling pathway in SH-SY5y cells. As such, we further examined whether enhanced HO-1 by THF treatment plays a critical role in its protective effects on METH-exposed SH-SY5y cells. Tin-protoporphyrin IX (SnPP) was used to remove induced HO-1 activity in SH-SY5y cells. As shown in Figure 7A, pretreatment with SnPP neutralized the cytoprotective effect of THF on cellular viability. To confirm whether HO-1 induced by THF is essential for the viability of SH-SY5y cells exposed to METH, the cellular confluency and intensity of annexin V and caspase 3/7 were assessed after pretreatment with SnPP. Figure 7B shows that the cellular confluency and intensity of caspase 3/7 were decreased compared to cells without pretreatment with SnPP. However, the intensity of annexin V was significantly increased by pretreatment with SnPP compared to that in cells without pretreatment with SnPP. To confirm whether HO-1 promoted by pretreatment with THF suppresses ROS production by METH exposure, the intensity of DCF-DA was compared between cells. Cells pretreated with SnPP and THF exhibited an increased intensity of DCF-DA compared to that in cells pretreated with THF alone (Figure 7C). These data suggest that the induction of HO-1 by pretreatment with THF mitigates METH-induced neurotoxicity.

### 2.8. Post-Treatment with THF Possesses a Protective Effect on METH-Induced Neurotoxicity

**Figure 8:**
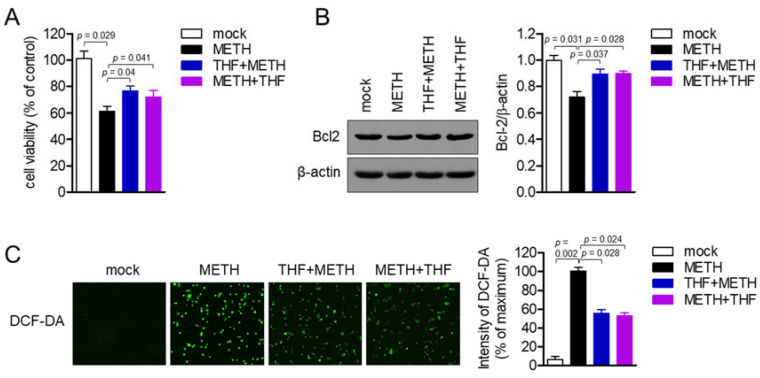
(**A**) SH-SY5y cells (1 × 10^4^/well, 96-well plate) were exposed to the 2 mM of METH for 24 h in the presence of pretreatment with THF or post-treatment with THF. Viability was measured by MTT assay. (**B**) SH-SY5y cells were exposed to the 2 mM of METH for 24 h in the presence of pretreatment with THF or post-treatment with THF. The level of Bcl-2 and β–actin was detected by western blot. Normalization was performed with the expression of Bcl-2 and presented in bar graph. (**C**) SH-SY5y cells (1 × 10^4^/well, 96-well plate) were exposed to 2 mM of METH for 24 h in the presence of pretreatment with THF or post-treatment with THF. Stained cells with 2 μM 2′,7′-dichlorofluorescin diacetate (DCF-DA) for 20 min were scanned to detect the production of reactive oxygen species (ROS) using the IncuCyte imaging system. The representative images were obtained and the intensity of DCF-DA was calculated as the percentage of the maximum. Results are shown as the mean value of three experiments ± SEM. *p* value between two indicated groups.

To investigate whether post-treatment with THF possesses a protective effect against METH-induced neurotoxicity, MTT viability assay was performed with cells pretreated and post-treated with THF, respectively. As shown in Figure 8A, post-treatment with THF also restored the cell viability in METH-exposed condition as pretreatment with THF did. The expression of Bcl-2 was also detected after post-treatment with THF following METH exposure. Figure 8B revealed that METH regulates the expression of Bcl-2 but post-treatment with THF slightly elevated as pretreatment with THF did. Figure 8C confirmed that post-treatment with THF modulates ROS production in SH-SY5y neuronal cells exposed to METH. These results suggest that post-treatment with THF also possesses a protective effect on METH-induced neurotoxicity as pretreatment with THF does.

## 3. Discussion

In the current study, we examined the protective effect of THF against METH-induced neurotoxicity in neuronal cells. Treatment with THF was found to not lead to cytotoxicity and apoptotic pathway activation up to a concentration of 40 μM, effectively reversing the viability of SH-SY5y cells that were reduced by METH exposure. The results from western blot and real-time PCR analyses showed that pretreatment with THF modulates apoptosis-related proteins and the oxidative stress generated by METH exposure in SH-SY5y cells. In this study, THF was found to induce Nrf2 nuclear translocation to produce the cytoprotective enzyme HO-1 via the PI3K/Akt/mTOR signaling pathway. The results from the inhibitor assay using SnPP showed that increased HO-1 activity was critical for the protective effect of THF treatment against METH-induced neurotoxicity in SH-SY5y cells. These data suggest that THF exerts a therapeutic effect against METH-induced neurotoxicity by regulating oxidative stress and enhancing HO-1 expression.

Several reports have demonstrated the protective bioactivity of the flavanone family derived from natural products against oxidative stress in neuronal cells. Flavanomarein, one of the main components of *Coreopsis tinctoria Nutt*., exerts protective effects against oxidative stress damage induced by 6-hydroxydopamine (6-OHDA) on PC12 and primary cortical neurons [32]. An experimental trial to investigate the beneficial effect of hesperetin, a flavanone family, on the neurotoxicity induced by 6-OHDA was also reported using a Parkinson’s disease animal model induced by injecting 6-OHDA [33]. In particular, flavanones isolated from citrus, including hesperidin, hesperetin, and neohesperidin, have been found to confer protection to PC12 neuronal cells against H_2_O_2_-induced cytotoxicity by reducing oxidative stress [34]. Here, we demonstrated that THF, a flavanone family, exerts an ameliorative effect against METH-induced neurotoxicity by suppressing oxidative stress and enhancing HO-1 expression in neuronal cells. These results suggest that small molecules from the flavanone family have the potential to be developed into therapeutic treatments for neurodegenerative disorders, including Alzheimer’s disease and Parkinson’s disease.

The caspase family has been shown to play a major role in the cell death pathway. In the caspase family cascade, caspase3 is located downstream and cleaved into active caspase3 during apoptosis. A recent report revealed that the PI3K/Akt/mTOR pathway is highly involved in the activation of caspase3 during the apoptotic pathway [35]. In brain tissue, the phosphorylation of PI3K/Akt/mTOR exerts an anti-apoptotic effect by maintaining caspase3 in its inactive form and improving neuronal damage [36]. In addition, Nrf2 is found downstream of the PI3K/Akt/mTOR signaling pathway under oxidative stress conditions [37]. A recent study has demonstrated that METH exposure enhances Nrf2 nuclear translocation into the nucleus for defense mechanisms by suppressing the phosphorylation of PI3K/Akt/mTOR in neuronal cells [38]. In the current study, we showed that pretreatment with THF reversed the phosphorylation of PI3K/Akt/mTOR (Figure 5) and blocked the cleavage of the caspase family, including caspase3 (Figure 3), in METH-exposed SH-SY5y cells. This suggests that THF regulates the phosphorylation of PI3K/Akt/mTOR by METH exposure and exerts a protective effect against apoptosis and cell death by reversing the caspase family. Future studies will be needed to determine whether treatment with THF activates the Nrf2 pathway in METH-exposed SH-SY5y cells compared to SH-SY5y cells without METH exposure.

## 4. Materials and Methods 

### 4.1. Cells

SH-SY5y neuronal cells (KCLB number: 22266) were obtained from the Korean Cell Line Bank (Seoul, Korea) and cultured in complete Dulbecco’s modified Eagle medium (DMEM) (Welgene, Gyeongsan, Korea) (1× penicillin G, 1× streptomycin, 10% fetal bovine serum (FBS), and 2 mM L-glutamine). Cells were passaged five to eight times prior to any experiments and cultured at 37 °C in a humidified incubator containing 5% CO_2_.

### 4.2. Preparation of THF from Dalbergia Odorifera

THF (C_15_H_12_O_5_) was isolated from *D. odorifera*, as previously reported [23]. Briefly, *D. odorifera* was obtained from the Herbal Medicine Cooperative Association of Jeonbuk Province, Korea. Dried *D. odorifera* (5 kg) was extracted four times using 100% ethanol. The filtered ethanol extract (0.604 kg) was concentrated and partitioned with CH_2_Cl_2_. The CH_2_Cl_2_-soluble fraction (50 g) was subjected to chromatography on a silica gel column with n-hexane-EtOAc (1:0 to 0:1) and five fractions (Fr. 1 to Fr. 5) were obtained. Among them, Fr. 3 (30 g) was separated with a mixture of solvents (MeOH:H_2_O = 9:1) on a Sephadex LH-20 column, and four subfractions were obtained (Fr. 3-1 to Fr. 3-4). Fr. 3-3 (7.5 g) was further purified with a mixture of solvents (EtOAc: MeOH = 4:1) on a Sephadex LH-20 column and loaded onto a silica gel column with a gradient mixture of solvents from 100% n-hexane to 100% EtOAc to obtain 15 subfractions (Fr. 3-3-1 to Fr. 3-3-15). Fr. 3-3-4 (30 mg) was characterized as THF by comparing the newly analyzed ^1^H and ^13^C nuclear magnetic resonance (NMR) (JNM-ECA 500, JEOL, Tokyo, Japan) spectral data with a previous report [39]. Using NMR spectroscopy, the purity was determined to be 98.8%.

### 4.3. Reagents and Antibodies

Annexin V staining reagents (cat. number: 4642) for the IncuCyte^®^ cell imaging system were obtained from Essen Bio (Ann Arbor, MI, USA). MTT, METH, DMSO, radioimmunoprecipitation assay (RIPA) buffer, DCF-DA, and SnPP were purchased from Sigma Chemical Co. (St. Louis, MO, USA). Antibodies against Bcl2 (cat. number: sc-7382), β-actin (cat. number: sc-47778), phosphorylated mTOR (cat. number: sc-293133), and Nrf2 (cat. number: sc-518033) were obtained from Santa Cruz Biotechnology (Dallas, TX, USA). Anti-caspase3 (cat. number: 9662), anti-caspase7 (cat. number: 9494), anti-Bax (cat. number: 5023), anti-SOD (cat. number: 2770), anti-CAT (cat. number: 14097), anti-phosphorylated PI3K (cat. number: 4228), anti-PI3K (cat. number: 4257), anti-phosphorylated Akt (cat. number: 4060), anti-Akt (cat. number: 9272), anti-mTOR (cat. number: 2983), and anti-LaminB (cat. number: 13435) were purchased from Cell Signaling Technology (Beverly, MA, USA). The NE-PER kit, PVDF membranes, and ECL detection reagent were obtained from Thermo Scientific (Rockford, IL, USA).

### 4.4. Determination of Confluency and Intensity of Annexin V

The cell confluency and intensity of annexin V and caspase 3/7 were assessed using the IncuCyte^®^ imaging system. Briefly, seeded SH-SY5y cells (5 × 10^3/^well or 1 × 10^4^/well, 96-well plate) were stained with 1× annexin V staining reagents and incubated under the indicated conditions. After incubation, adherent cells were marked in yellow and fluorescence was scanned using the IncuCyte^®^ imaging system. The cell confluency and intensity of annexin V were automatically obtained and the percentage of maximum was calculated by dividing the intensity of experimental group with the maximum intensity. These percentages were presented as bar graphs.

### 4.5. MTT Viability Assay

After incubating the SH-SY5y cells (5 × 10^3^/well or 1 × 10^4^/well, 96-well plate) under the indicated conditions, MTT (500 μg/mL) was added to the cells for 2 h. The supernatants were removed and 150 μL of dimethyl sulfoxide was added to the wells to dissolve the formazan crystals formed at the bottom of the wells. The plate was read at 540 nm to obtain the OD values, which was used to calculate the cell viability as a percentage of the control.

### 4.6. Western Blot Analysis

The SH-SY5y cells were incubated at the indicated conditions, harvested, and lysed for western blot analysis using RIPA buffer. After lysing for 30 min on ice, the cells were centrifuged at 14,000 rpm for 15 min at 4 °C. The resulting supernatants were collected for further analysis. Bradford assay was performed to measure the concentration of lysate, and approximately 30–40 μg of lysate was loaded onto 8–12% sodium dodecyl sulfate (SDS)-polyacrylamide gel electrophoresis (PAGE) gels. After protein separation, whole proteins were transferred onto PVDF membranes and blocked with blocking buffer (5% skim milk) for 1.5 h at room temperature (RT). After three rinses with Tris-buffered saline containing 0.1% Tween-20 (TBS-T), primary antibodies (dilution factor was 1 to 1000) in 1% skim milk in TBS-T (dilution factor, 1:1000) were added to the membranes overnight at 4 °C. After incubation, the excess primary antibodies were discarded with three rinses with TBS-T and 0.1 μg/mL peroxidase-labeled secondary antibodies (against rabbit or mouse, dilution factor was 1 to 5000) were added to the membranes for 1 h. After three rinses with TBS-T, ECL detection reagents (Thermo Fisher Scientific) and ImageQuant LAS 4000 (GE Healthcare, Chicago, IL, USA) were used to visualize the specific bands. Every detected band was normalized to the loading controls (β-actin for whole lysate or cytosolic extracts and LaminB for nucleic extract) and presented as bar graphs.

### 4.7. Quantitative Real-Time PCR Analysis

To assess the mRNA levels of the indicated genes in the SH-SY5y cells, TRIZOL reagent was used to isolate total RNA, and RT PreMix (Enzynomics, Daejeon, Korea) was used for reverse transcription. The DNA Engine Opticon 1 continuous fluorescence detection system (MJ Research, Waltham, MA, USA) was used for quantitative real-time PCR analysis using SYBR Premix Ex Taq (Takara, Japan). The total reaction volume was 10 μL, containing 0.1 μg of cDNA and gene-specific primers. The primers used in this study were as follows (forward and reverse primers, respectively): human SOD, 5′-CTC ACT CTC AGG AGA CCA TTG C-3′ and 5′-CCA CAA GCC AAA CGA CTT CCA G-3′; human CAT, 5′- CTT GGA ACA TTG TAC CCG CT-3′ and 5′-GTC CAG AAG AGC CTG AAT GC-3′; human HO-1, 5′-CCA GGC AGA GAA TGC TGA GTT C-3′ and 5′-AAG ACT GGG CTC TCC TTG TTG C-3′; human GAPDH, 5′-CGG AGT CAA CGG ATT TGG TCG TAT-3′ and 5′-AGC CTT CTC CAT GGT GGT GAA GAC-3′. Each PCR reaction was performed under the following conditions: 95 °C for 30 s, 60 °C for 30 s, and 72 °C for 30 s, plate reading (detection of fluorescent product) for 40 cycles, followed by 7 min of extension at 72 °C. A melting curve was obtained by gradually increasing the temperature (0.2 °C/s) from 60 °C to 95 °C with a collection of fluorescence data at 0.2 °C intervals to characterize the dsDNA product. The percentage of the maximum was calculated using the following equation: % of maximum = 2^−ΔΔCT^ × 100, where ΔΔCT = (CT_target_ − CT_gapdh_) at maximum − (CT_target_ − CT_gapdh_).

### 4.8. Determination of ROS Production Using IncuCyte Imaging System

SH-SY5y cells (1 × 10^4^/well, 96-well plate) were incubated under the indicated conditions and stained with 2 μM DCF-DA for 20 min at 37 °C in the dark. The generated fluorescence was scanned using the IncuCyte imaging system. The intensity of DCF-DA was calculated as a percentage of the maximum and presented in bar graphs.

### 4.9. Determination of Nrf2 Nuclear Translocation

For the separation of nucleic extracts from whole lysates, the NE-PER kit was used according to the manufacturer’s instructions. Briefly, incubated SH-SY5y cells under the indicated conditions were harvested and lysed using lysis buffer I. After centrifugation with lysis buffer II, the lysate was removed for further analysis (cytosolic extract). Pellets were lysed using lysis buffer III and centrifuged to isolate the nucleic extracts. Approximately 30 μg of lysate was loaded onto 6% SDS-PAGE gels. The expression of LaminB and β-actin indicates the purity of the nucleic and cytosolic extracts, respectively. The detected Nrf2 was normalized to the expression of LaminB (nucleic extract) and β-actin (cytosolic extract).

### 4.10. Statistical Analysis

One-way analysis of variance was used to assess the significance (*p*-value). The mean values ± SEM were calculated from the data of three independent experiments performed on separate days, presented in graphs with *p* values.

## Data Availability

The data presented in this study are available on request from the corresponding author.

## Supplementary Materials

The following are available online. Figure S1. Exposure to METH leads to neurotoxicity in dose-dependent manner.

## Sample Availability

Not available.
