# Supplementary material for: 6,7,4′-Trihydroxyflavanone Mitigates Methamphetamine-Induced Neurotoxicity in SH-SY5y Cells via Nrf2/heme Oxyganase-1 and PI3K/Akt/mTOR Signaling Pathways
Source: Molecules. 2021 Apr 22;26(9):2442. doi: 10.3390/molecules26092442 (PMC8122742; doi:10.3390/molecules26092442)

Figure S1. Exposure to METH leads to neurotoxicity in dose-dependent manner.

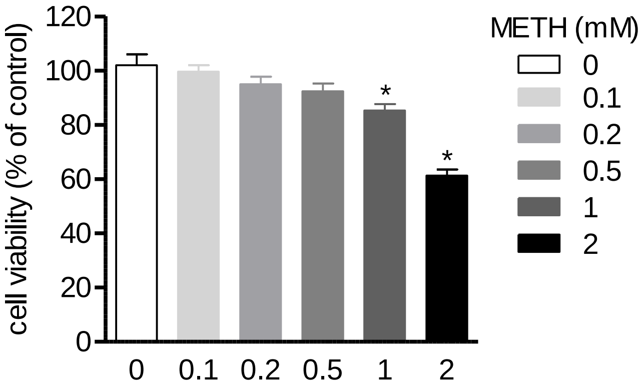

Figure 3A

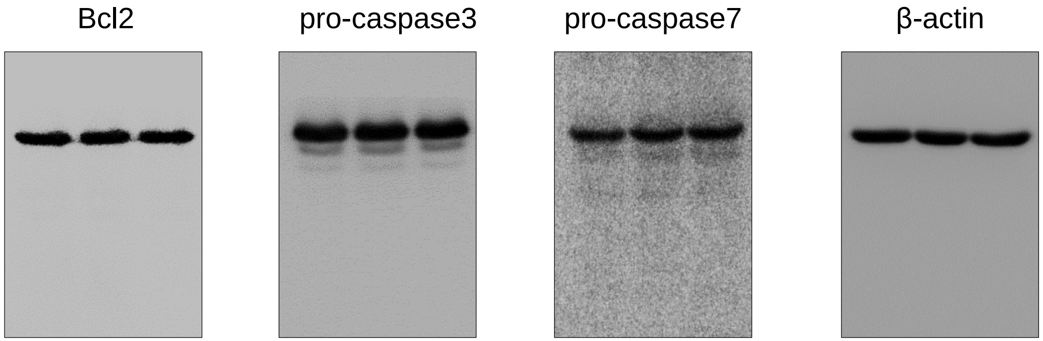

Figure 3C

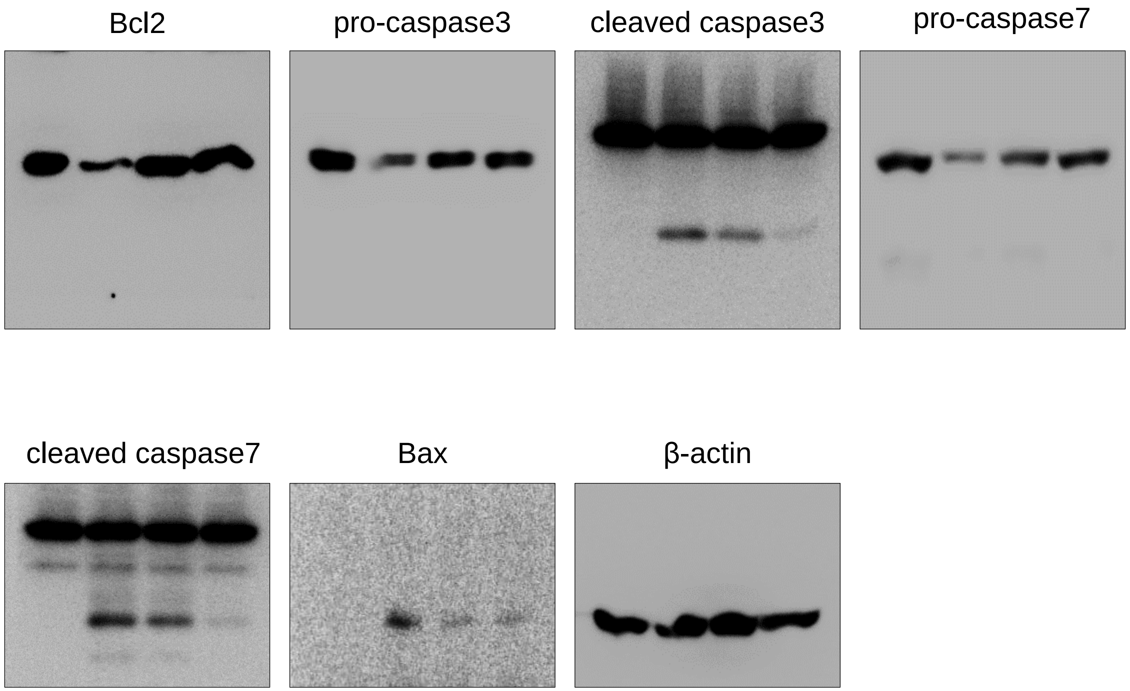
Bcl2
pro-caspase3
cleaved caspase3
pro-caspase7
cleaved caspase7
Bax
β-actin

Figure 4C

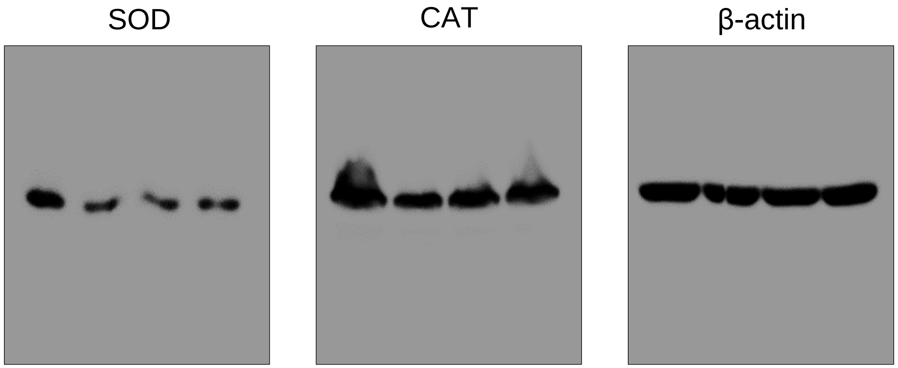

Figure 5A

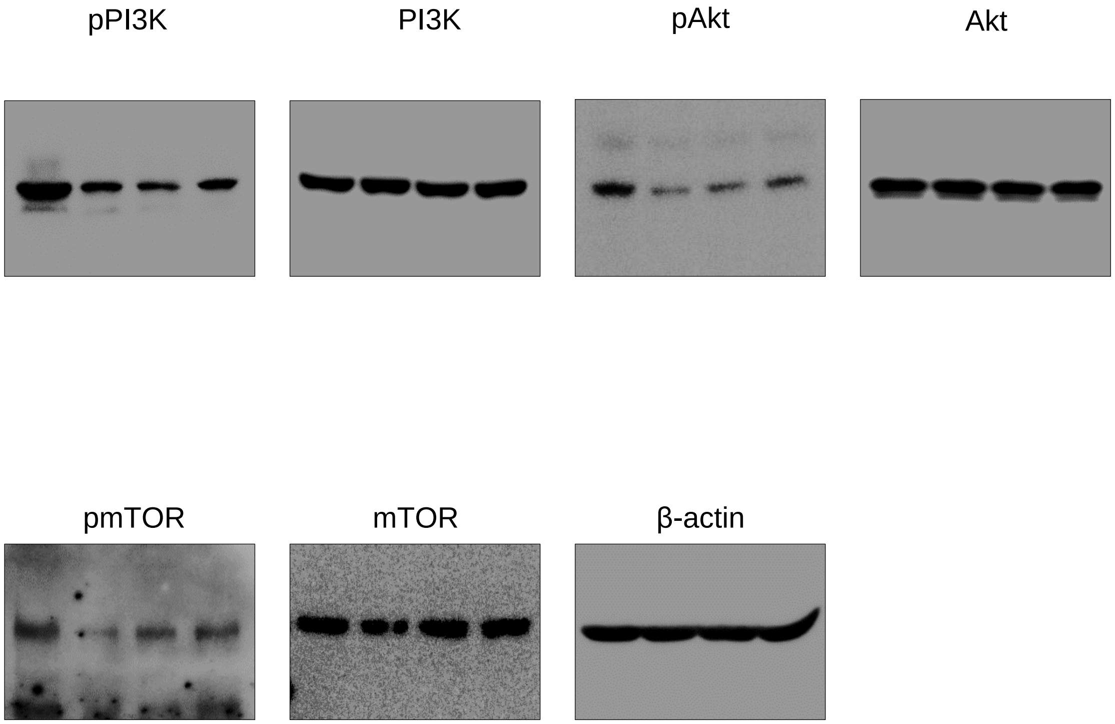

Figure 5C

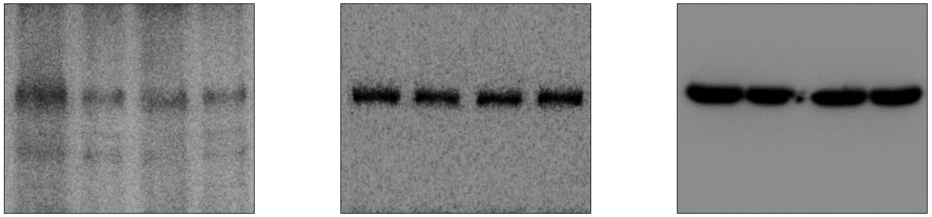

Figure 6A

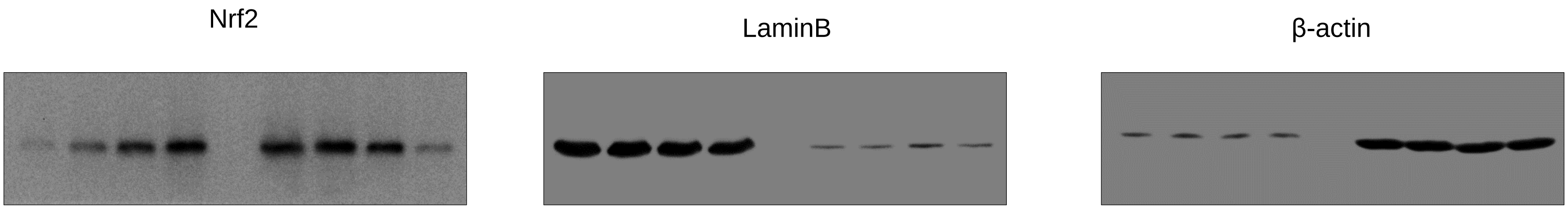

Figure 6B

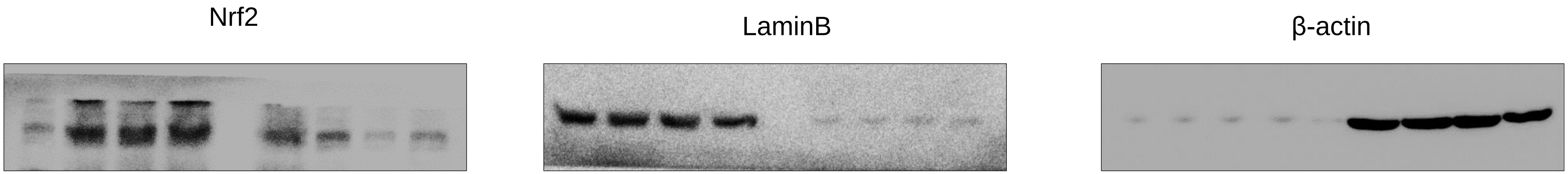

Figure 8A

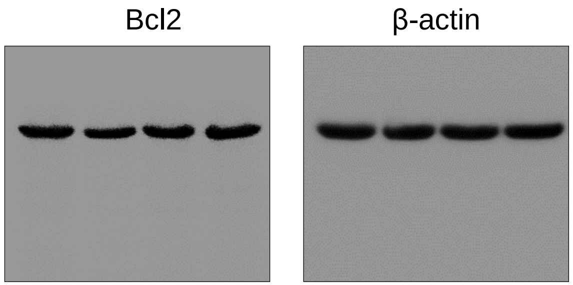

Supplement: Supplementary file 1 [file molecules-26-02442-s001.zip › molecules-1146401-SI.pdf]
